# Seedling Discrimination with Shape Features Derived from a Distance Transform

**DOI:** 10.3390/s130505585

**Published:** 2013-04-26

**Authors:** Thomas Mosgaard Giselsson, Henrik Skov Midtiby, Rasmus Nyholm Jørgensen

**Affiliations:** Institute of Chemical Engineering, Biotechnology and Environmental Technology, University of Southern Denmark, Campusvej 55, DK-5230 Odense, Denmark; E-Mails: hemi@kbm.sdu.dk (H.M.); rasj@kbm.sdu.dk (R.J.)

**Keywords:** object recognition, machine vision, shape characterization

## Abstract

The aim of this research is an improvement of plant seedling recognition by two new approaches of shape feature generation based on plant silhouettes. Experiments show that the proposed feature sets possess value in plant recognition when compared with other feature sets. Both methods approximate a distance distribution of an object, either by resampling or by approximation of the distribution with a high degree Legendre polynomial. In the latter case, the polynomial coefficients constitute a feature set. The methods have been tested through a discrimination process where two similar plant species are to be distinguished into their respective classes. The used performance assessment is based on the classification accuracy of 4 different classifiers (a k-Nearest Neighbor, Naive-Bayes, Linear Support Vector Machine, Nonlinear Support Vector Machine). Another set of 21 well-known shape features described in the literature is used for comparison. The used data consisted of 139 samples of cornflower (Centaura cyanus L.) and 63 samples of nightshade (Solanum nigrum L.). The highest discrimination accuracy was achieved with the Legendre Polynomial feature set and amounted to 97.5%. This feature set consisted of 10 numerical values. Another feature set consisting of 21 common features achieved an accuracy of 92.5%. The results suggest that the Legendre Polynomial feature set can compete with or outperform the commonly used feature sets.

## 1. Introduction

Development in legislations, taxations and environmental awareness has led the agricultural industry to follow a “greener” line. The shift requires that machinery has to be changed and management practices need to be updated. However, it is a necessity to maintain the economical feasibility of agriculture. For farmers, cultivating their fields, this means shifting towards using fewer chemicals. For many years, researchers have investigated methods to help farmers achieving the goals of less chemical use by updating or inventing new equipment capable of performing needed tasks such as weeding in a more environment-friendly and cost-effective way. In general, precision weed management systems consist of a subsystem capable of detecting and identifying plants, then information is fed to a decision subsystem that makes an informed management decision, and finally a subsystem execute the management decision. This paper is focused on an essential part of the subsystem that identifies plants. The basic idea is that knowing the positions of individual plants and their species will enable treatment either mechanically or chemically on a single plant level. Several researchers and companies are working on this kind of technology. The approaches vary from remote sensing to individual plant inspection or crop mapping using positioning systems as described in the 2005 review paper by Brown and Noble [1]. In this paper, camera technology is combined with object discrimination and classification by shape. The image analysis task is highly non-trivial because it deals with outdoor scenes and the objects under consideration deform and vary their appearance according to a number of external factors, such as time of day, general soil conditions, weather conditions and season. Furthermore, acquisition specific constraints such as viewing angle, occlusion and light conditions affect the image quality and thereby increase the complexity of the analysis task.

Before shapes can be analysed in images, preprocessing needs to be performed. Often images are recorded by commercially available cameras that quantify colour into red, green, and blue (RGB) intensities. This is the approach taken in this paper. Woebbecke *et al.* [2] reviewed and investigated different mappings of RGB colour in order to qualify how to achieve a best possible linear separation of green plant material and soil material. The conclusion was that a linear combination of the form 2g−r−b, with the chromatic values r, g and b or a modified hue value, possessed the best separation capability between soil/residue and green plant material. They also concluded that none of the investigated mappings were able to robustly distinguish between monocotyledonous and dicotyledonous plants.

Meyer and Neto [3] also examined different mappings of chromatic values of RGB images and included an investigation of the effect of varying background material. One conclusion of their work was that when images consisted of soil as background and green plant material as foreground, then ExG (Excessive Green) with an automatic Otsu threshold [4] and ExG-ExR with ExR being Excessive Red (boils down to G-R) with a static threshold of 0 performed equally well—both achieved a score of approximately 0.87 calculated by dividing the true positives with (true positives + false negatives + false positives).

After the preprocessing step, objects of green vegetation can be extracted from the images. Several researchers have been trying to classify blobs of connected components into species categories or other pre-defined groups.

As a follow-up on their work with plant/background segmentation, Woebbecke et al. [5] investigated shape features that can be used for discriminating whole blobs of either a monocotyledonous plant species or a dicotyledonous plant species. Their work included three dicotyledonous species and nine monocotyledonous species. Result from data analysis indicated that before the age of 14 days after emergence, the shape feature referred to as aspect was best at distinguishing between monocotyledonous and dicotyledonous plants (accuracy of 74%), whereas the shape feature *ICM_1_* (first invariant image moment) performed the best after age 14 days (accuracy of 89%).

Slaughter and Giles [6] described a system distinguishing between tomato leaves and weed leaves by calculating the elongation and compactness of connected components. They reported that among a large number of shape features, elongation and compactness proved to be sufficient. For the classification part, they regarded plants as belonging to one of four classes: tomato cotyledon, tomato true leaf, miscellaneous tomato leaf and weed. They achieved an overall classification accuracy of 73.1% correctly classified tomato leaves and 68.8% correctly classifies weeds.

Hemming and Rath [7] constructed a system for discriminating crops from weeds. They conducted green house and field test where the crops were either cabbage (*Brassica oleracea* L.) or carrot (*Daucus carota* L.). Classification was accomplished by using eight form features and three colour features. Not all features were necessarily used, since they also conducted feature selection and feature weighting. They reported a mean classification accuracy of 88.15% for cabbage and 72.46% for carrots.

In [8] Weis and Gerhards investigated the performance of several shape features for categorizing plants into groups. They state that their feature analysis was based on a feature set of over 100 numerical features, including some novel features based on object skeleton and distance transform. Through feature reduction, region-based and skeleton-based features were found to perform the best and ranked higher than contour-based features such as Fourier descriptors and curvature scale space representations. Like other studies, Weis and Gerhards also focused on object class characterization. Often object characterization is regarded as dividing objects into groups in terms of species. Weis and Gerhards worked with groups defined by species, growth stage and geometric category (single leaf, whole plant, overlapping). They reduced the amount of classes by grouping species that had similar structure or were sensitive to the same herbicide. In a case study consisting of 568 samples distributed among 4 class groupings, they reported a classification accuracy of 98.6%.

Slaughter *et al.* published in 2008 a review paper [9] about plant species recognition methods by ground-based machinery. They divide the methods into 3 categories based on the approach, namely: biological morphology, spectral characteristics, and visual texture. They define morphology as being shape and structure or topology and they note that often recognition is done at leaf level rather than at whole plant level. Several systems are described, but they argue that because of problems handling defects, occlusion, soil splash and other appearance-changing factors, no robust commercial system is currently available.

Another review article by López-Granados [10] also discussed the state of plant species recognition. One point that he made is that within-class differences are huge and he concluded that a robust recognition system will need to be very complex.

The complexity of features can be assessed by their level of invariance. Well performing generic shape features used in computer vision often possess three properties of invariance, *i.e.*, their ability to be invariant to scale, rotation and translation. With such properties and a robust object extraction method, objects can be recognised as long as they are not subject to affine or projective transformation. If an object is only subject to similarity transformations, its values will not change, if features obey stated invariance properties. Some literatures claim to construct features that are also invariant to small affine transformations[11,12], which suggests that they are also more robust in terms of other variations.

Regarding invariance, one thing to consider for this case study is that the need for scale invariance arises not from camera distance changes, since it is expected to be fixed, but as a consequence of plants being at different growth stages. Furthermore, the objects considered in this case study are not rigid, such that the previously described feature invariances are only minimum requirements. Ideal features would be invariant to natural variations of an object class. The existence of such natural invariant features is unknown to the authors.

In this paper we propose two novel feature sets describing the shape of objects by parametrising the distribution of distance values calculated by conducting a distance transform. Feature generation is achieved with two approaches. The first approach applies a distance transform and extracts distances at specific places. The second approach approximates the distance distribution with Legendre polynomials and extracts its coefficients. For both methods a variable number of features could be generated but in this work we chose to limit the investigation to 10 numerical features for each set.It is believed that the idea of parametrising the result of a distance distribution of non-rigid objects has not been done before. After the feature generation step, a classification step followed. Four classifier models were applied: k-Nearest Neighbor, Naive Bayes, Linear Support Vector Machine and Non-linear Support Vector Machine. An early version of the feature generation methods used in this paper is described in the master thesis of the first author [13].

The paper is partitioned as follows. Section 2 documents the data processing from image acquisition to classification results. Section 2.1 describes the acquisition setup and preprocessing steps specifically. Section 2.4 describes and lists features reported in the literature that were used for comparison with the proposed feature set. Subsection 2.2.2 describes the data mapping and preprocessing prior to feature extraction. Subsection 2.2.3 describes the two mentioned approaches to feature generation. Section 2.5 describes the used classifiers and classification tests. Section 3 presents the result of the current work followed by a discussion and conclusions in Sections 4 and 5.

## 2. Materials and Methods

### 2.1. Used Data

**Figure 1.**
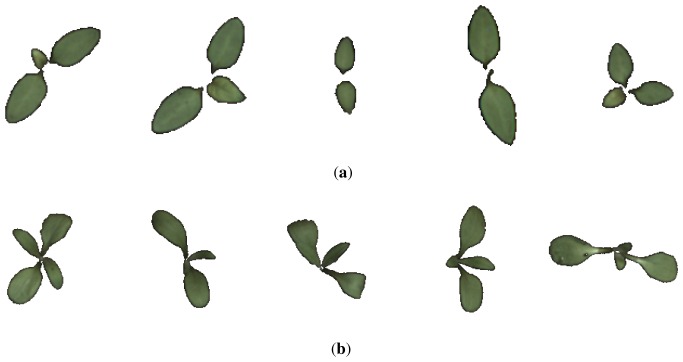
10 random samples from the image database. (**a**) 5 random nightshade samples from the image database; (**b**) 5 random cornflower samples from the image database.

The collected data consisted of 139 image samples of cornflower (*Centaurea cyanus*) and 63 image samples of nightshade (*Solanum nigrum*). Both plant species were in a growth stage BBCH 12 [14]. Sample images of the two species are shown in Figure 1.

#### 2.1.1. Data Recording

**Figure 2.**
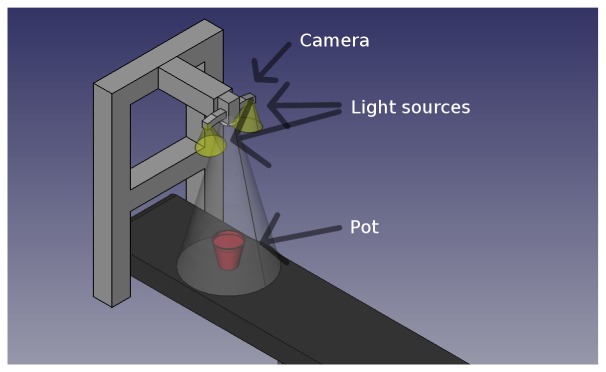
Sketch of recording camera setup and light source placement.

**Figure 3.**
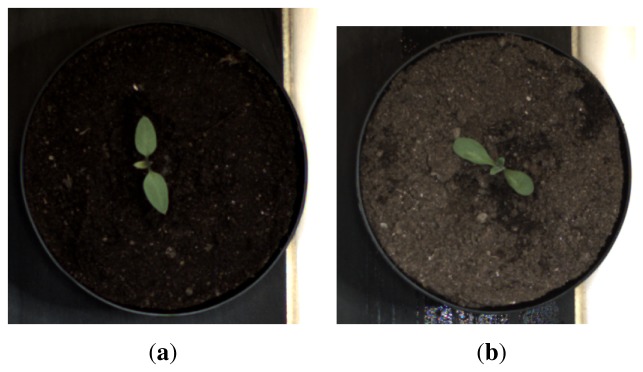
Raw sample images. (**a**) Raw nightshade sample; (**b**) Raw cornflower sample.

Images were acquired indoors under controlled light conditions with an RGB CMOS camera (PixeLINK PL-B742F-R) with a resolution of 1,280 × 1,024. Each plant was sown in its own pot to facilitate tracking of plants and minimize plant overlap. The pots were put on a conveyor belt (see Figure 2) and moved past a down facing camera. When the plant was beneath the camera an image was recorded and stored together with the species name and growth stage. Figure 3 shows two examples from the image data base.

Figure 2 illustrates the setup. The camera was mounted 80 cm above the conveyor belt, which gave a physical resolution of 2.9 pixels per millimetre in the recorded images. Two light sources consisting of multiple LEDs were mounted close to the camera to prevent hard shadows.

#### 2.1.2. Preprocessing

To extract the actual plant objects in the images, noise was removed by applying a 3 × 3 Gaussian filter, then underexposed regions were detected and marked as unreliable with respect to colour information. The remaining pixels that were not marked as unreliable were then colour transformed and lastly a segmentation was conducted based on a region growing approach [13].

The colour transformation consisted of calculating the excess green (ExG) value for each pixel [2]. This approach was chosen according to Woebbecke et al. in [2]. After a binarisation process, plants could be extracted, often as a single connected component. To ensure comparable shape features, it was required that every plant consisted of a single connected component. If this was not the case after the binarisation, an artificial rejoining process was conducted as described in [13]. This process consisted of finding the closest points on the contour of two objects that are not connected. Based on the located points, objects are joined by a small strip and the two objects become connected. If a plant consists of more than two objects, the two objects closest to each other are found and joined and the process is repeated.

### 2.2. Proposed Feature Sets

In this paper, two new feature sets are described. They are both calculated from contours of objects, but nevertheless allow object parts to be disconnected. Both feature sets are based on a distance transform of those contours. The first step in the feature generation process consists of data generation and mapping to achieve a new data structure that eases feature extraction. This preprocessing is described in the following subsections.

#### 2.2.1. Distance Transform

**Figure 4.**
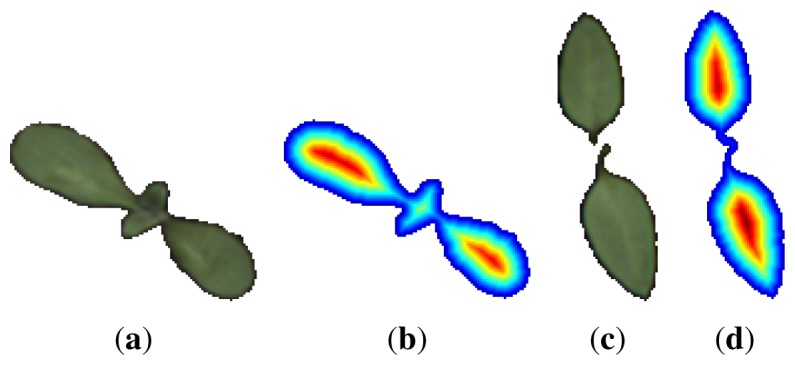
Data set samples and their associated distance transforms. (**a**) Segmented cornflower sample; (**b**) Segmented nightshade sample; (**c**) Distance transform of cornflower sample; (**d**) Distance transform of nightshade sample. Note that two components have been rejoined. Blue equals small distance, red equals largest distance.

The distance map or transform, *M_map_*, is a matrix of same dimensions as the binary image containing the object under consideration. Each matrix element corresponds to one pixel in the image. Every element that corresponds to an object pixel contains the distance, *d*, to the nearest edge of the object; all other matrix elements are set to −1. The distance measure used in this work is the true Euclidean distance. Figure 4 shows a nightshade and a cornflower sample and their associated distance transforms.

A distance vector is obtained by collecting all distances *d* ≠ −1 in the matrix *M_map_* into a single vector, $D_{\text{vector}}^{\to }$, in an unordered fashion. $D_{\text{vector}}^{\to }$ constitutes the distance distribution of the current object. The vector $D_{\text{vector}}^{\to }$ will not be invariant to rotation of the original object but translation invariance is ensured through the nature of the distance transform. To ensure rotation invariance, the vector $D_{\text{vector}}^{\to }$ has been sorted making a new vector $D_{\text{sort}}^{\to }$.

#### 2.2.2. Data Mappings

**Figure 5.**
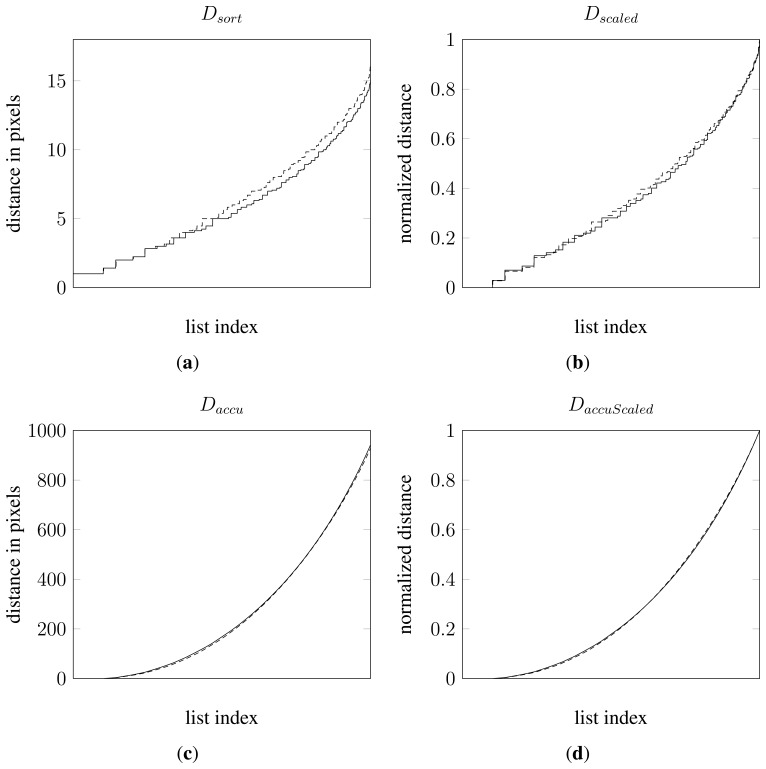
Comparison of the four data mappings for the nightshade and cornflower samples in Figure 4. Dashed lines originate from the nightshade sample and solid lines from the cornflower sample. (**a**) plot of sorted distances; (**b**) plot of sorted and scaled distances; (**c**) plot of sorted and accumulated distances; (**d**) plot of sorted, accumulated and scaled distances.

From $D_{\text{sort}}^{\to }$, three additional vectors were constructed by applying linear mappings and accumulation—all with the purpose of improving the data representation. The only basic invariance criterion not fulfilled is scale invariance. As mentioned in the introduction, scale differences arise for plants being at different growth stages.An analytic approach to eliminate scale has not been attempted. Instead, a normalization has been applied. By normalising $D_{\text{sort}}^{\to }$ with the maximum observed distance in an object, a new vector was formed referred to as $D_{\text{scaled}}^{\to }=\frac{D_{\text{sort}}^{\to }}{D_{\text{sort}}^{\to }[N]}$, where *N* denotes the number of elements in the vector. Another mapping based on $D_{\text{scaled}}^{\to }$ was constructed by calculating the accumulated sum of the elements in $D_{\text{scaled}}^{\to }$. This led to a vector referred to as $D_{\text{accu}}^{\to }[n]=\sum_{i=1}^{n}D_{\text{sort},i}^{\to }$ for *n* = 1…*N*. The last mapping consisted of normalised values of $D_{\text{accu}}^{\to }$ constructed in the same way as $D_{\text{scaled}}^{\to }$ by normalizing with the greatest value in the vector. This last vector will be referred to as $D_{\text{accuScaled}}^{\to }=\frac{D_{\text{accu}}^{\to }}{D_{\text{accu}}^{\to }[N]}$. All together a total of 4 vectors or data mappings were tested. Figure 5 shows 4 plots, one for each data mapping of the distance data from the two samples in Figure 4.

#### 2.2.3. Feature Generation

The proposed feature sets in this paper parametrise the formerly mentioned four vectors. Two methods were tested. The first method is a simple data re-sampling that is cheap to compute. The second method approximates the data with a 9 order Legendre polynomial.

**Figure 6.**
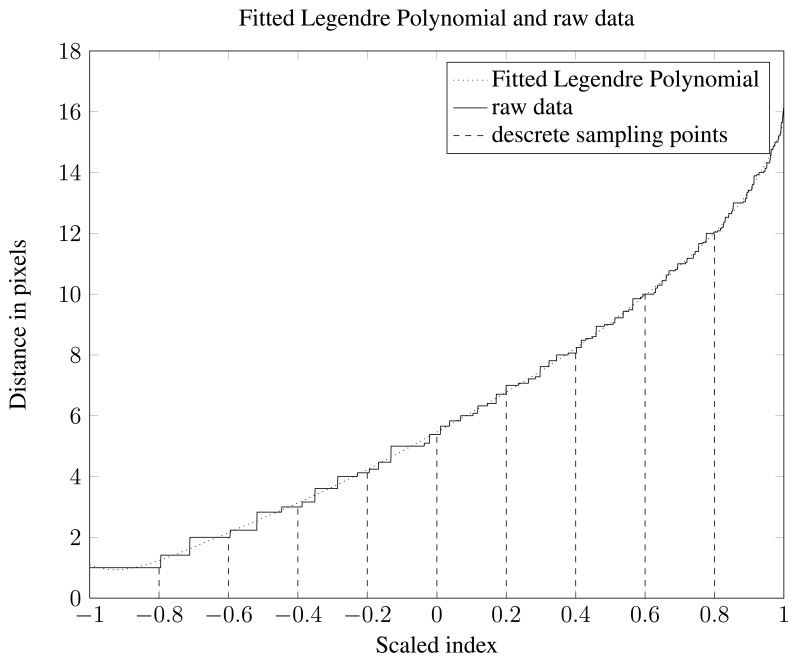
Visualization of data together with a fitted 9 order Legendre polynomial and descrete sampling points.

Re-sampling the data sets was done by determining 11 equidistant indices that cover the whole vector of distance data from a single object. The first one of these 11 is discarded since it will always be the smallest possible distance between an object boundary and an object pixel. This leaves 10 index values. Extracting the distance values at the 10 index positions finishes the feature generation step. A visualization of this process is shown in Figure 6. The ten distances are used as features and are referred to as the Re-Sampled Feature Set (*RSFS*).

As with the resampling method, the fitting of a polynomial has the purpose of parametrising the resulting distribution of the data mappings. Compared with the resampling method, the approximation of data by a function means that approximately the same information is stored in a different way. In this paper, a 9th order polynomial has been chosen as the function to approximate to data. It will later be investigated if the chosen order is appropriate by conducting feature selection and analysing the performance of several feature subset. The polynomial *P*(*x*) is constructed by the weighted sum of the first 10 Legendre polynomials ([15][p. 302]):

(1) $$P(x)=\sum_{n=1}^{10}a_{n}p_{n}(x)$$

where *p_n_*(*x*) is a n − 1 order Legendre polynomial. The Legendre polynomials are orthogonal with respect to the inner product:

(2) $$\langle p_{i}(x),p_{j}(x)\rangle =\int_{-1}^{1}p_{i}(x)p_{j}(x)dx$$

where 〈•, •〉 means the inner product and obey

(3) $$\langle p_{i}(x),p_{j}(x)\rangle =0\;\text{for}\;i\neq j$$

Choosing *p*_1_(*x*) = 1 and *p*_2_(*x*x) = *x*, a three term recursion formula can be formulated to calculate additional polynomials

(4) $$p_{n+2}(x)=[(2\cdot n+1)\cdot x\cdot p_{n+1}(x)-n\cdot p_{n}(x)]\frac{1}{n+1}$$

Equations 5–8 contains the first 4 Legendre polynomials.

(5) $$p_{1}(x)=1$$

(6) $$p_{2}(x)=x$$

(7) $$p_{3}(x)=\frac{1}{2}(3x^{2}-1)$$

(8) $$p_{4}(x)=\frac{1}{2}(5x^{3}-3x)$$

The discrete data of the form $\left(\vec{x},\vec{y})=([\vec{x_{i}}],[\vec{y_{i}}]\right)$ for *i* = 1… *N*, where *N* is number of data points, was approximated with an *α* order Legendre polynomial. Coefficients for each constituting Legendre polynomial have been estimated. This can be done with the formula

(9) $$a_{n}=\frac{\langle p_{n}(\vec{x}),\vec{y}\rangle }{\langle p_{n}(\vec{x}),p_{n}(\vec{x})\rangle }$$

The 〈*p_n_*(*x⃗*), *p_n_*(*x⃗*)〉 term can be written as $\frac{n}{2n-1}$.

The argument for choosing to approximate Legendre polynomials instead of ordinary polynomials lies in the fact the coefficients of the Legendre polynomial are uncorrelated whereas in ordinary polynomials the coefficients are correlated. This gives the advantage that if an investigation of the polynomial approximation reveals that some coefficients are irrelevant, these coefficients can be excluded from the coefficient calculation without affecting the calculation of other coefficients.

To do fitting with a Legendre polynomial data needs to be rescaled so that the x values span the interval [−1,1]. Visualization of data together with a fitted 9 order Legendre polynomial can be seen in Figure 6. A feature set consisting of polynomial coefficients was generated and is referred to as Legendre Polynomial Feature Set (*LPFS*).

### 2.3. Feature Calculation Complexity

As argued earlier, the *RSFS* method is computationally less expensive compared with the *LPFS* method. This is evident when analysing the computational complexity of the two methods. Complexity is first analysed with respect to number of pixels, *q*, in one image according to the currently used implementation. Table 1 lists the complexity of the preprocessing steps.

Each object was analysed individually after preprocessing. The complexity from this point on is with respect to the number of pixels, *m*, contained in one connected component and is according to the current implementation. Table 2 lists the complexities.

The *RSFS* and the *LPFS* method share the first 3 steps of the connected component processing and then they diverge, where the *RSFS* method does resampling and the *LPFS* method does polynomial fitting. The resampling process consists of extracting a fixed number of discrete samples and is therefore not dependent on the number of pixels. The LPFS method has an extra processing step that adds *O*(*m* × *order*) to the overall processing complexity compared with the *RSFS* method.

### 2.4. Common Shape Features

For comparison purposes, 21 well known contour and region features were computed. They were all derived from closed contours or regions of plant object. Below is a list of used features.

- 7 invariant image moments (Hu moments) [16]
- Object area
- Object perimeter
- Convex hull area
- Eccentricity [17]
- Solidity [18]
- Convex hull perimeter
- Perimeter ratio [17]
- Compactness [19]
- Circular variance [20]
- Elliptic variance [20]
- 4 skeleton based features [8]

Features with no reference in the above list are described in the review paper by Zhang and Lu [21]. These features are a subset of the typical shape features used for plant classification in the literature [5,8,16–21]. This feature set will be referred to as the Common Feature Set (CFS). The features are used to train classifiers for the purpose of comparison with classifiers trained with the proposed *RSFS* and LPFS feature sets.

### 2.5. Feature Set Quality Assessment

The quality of the proposed feature sets is assessed by the performance of classifiers using each of the proposed feature sets, RSFS and LPFS, and compared with the results of a classifier based on a common shape feature set CFS. To avoid numerical problems, the feature values were translated and scaled to the interval [−1,1] before using in any classifier. Four different classification models were used in the comparison: A k-Nearest neighbour, a Naive Bayes, a linear Support Vector Machine (SVM) and a Radial-basis Function Support Vector Machine (RBF-SVM). All experiments were carried out by using the classification model implementations in the MATLAB Toolbox PRTools [22]. The performance of each classifier is measured by their accuracy.

(10) Accuracy=(TP+TN)/N

where *TP* = true positives, *TN* = true negatives and *N* = total number of samples. All of the classifiers are supervised learning algorithms and need a training set. The division of the samples into a training and a test set was accomplish by stratified cross validation to ensure that no samples are used both for training and testing and that the class distribution in the two sets are alike. The cross validation was performed using 5 folds.

## 3. Results

This section documents and discusses achieved classification results and tries to characterise the errors done by the best performing classifier.

### 3.1. Classification Results

**Figure 7.**
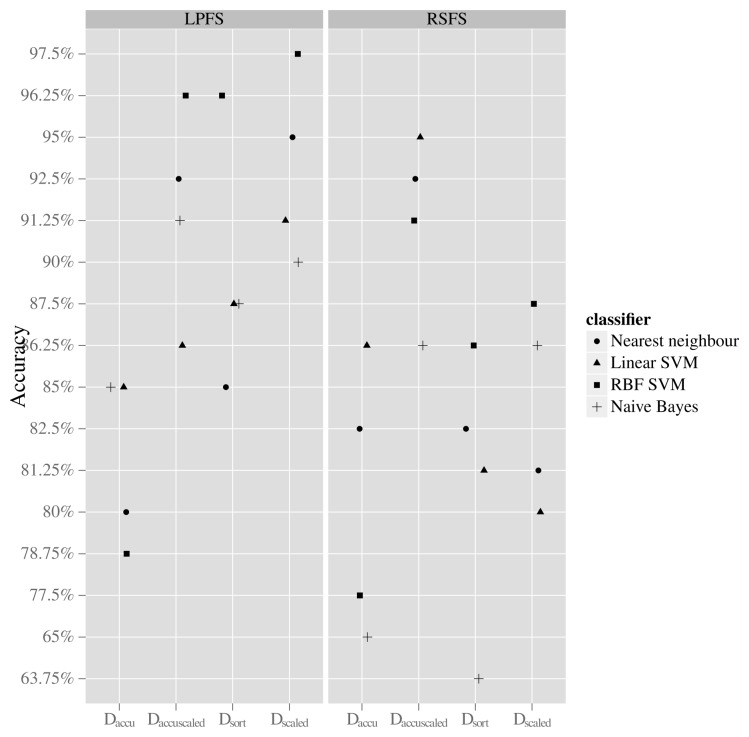
Classification results of using 4 different classifier models on 4 different data mappings using the RSFS and the LPFS method.

A total of 36 classification results were initially collected. This number emerges from having four data mappings, two feature generation methods and four classifiers plus additional four since each classifier also was used with the CFS feature set. The results can be seen in Figure 7 and Table 3. The data mapping that resulted in the highest average classification accuracy across all used classifiers is the *D_accuScaled_*. When using this mapping, the accuracy of all classifiers is retained in the interval from approximately 86% to 96% regardless of the used feature generation method. When using the mapping *D_scaled_*, the accuracy of the classifiers lies in the interval from 90% to 97% when using the LPFS feature generation method, whereas the accuracy is in the interval of 80% to 87% when using the RSFS feature generation method. The best result is achieved when using the *D_scaled_* mapping and the LPFS method for generating features and letting an RFB-SVM classifier conduct classification, giving an accuracy of 97.5%.

### 3.2. LPFS Feature Distributions

**Figure 8.**
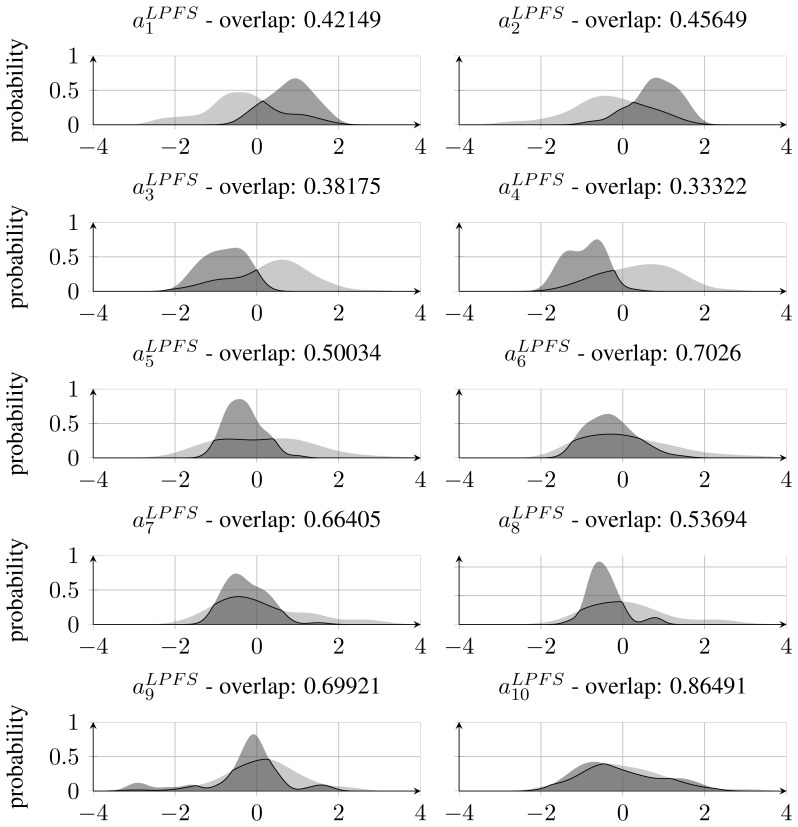
Distribution of the LPFS feature set generated from *D_scaled_* for both nightshade (light grey) and cornflower (dark grey). For each subfigure the amount of overlap between distributions is stated.

As stated in Section 3.1, the best performing feature set was found to be the LPFS features calculated on the *D_scaled_* mapping. When inspecting normalised feature distributions of each of the features that constitute this set, it becomes clear that features corresponding to the coefficients of the polynomial above the 5th degree have relatively more overlap between the classes compared with those below 6th degree. This can be seen in Figure 8, which also states the amount of overlap. The high overlap indicates that it might be possible to remove those features and thereby reduce the feature space without losing significant classification accuracy. This has been investigated by training and testing a RBF-SVM classifier with only the first 5 coefficients of the fitted Legendre polynomial. This resulted in an accuracy of 90.6%, which is regarded as a significant drop from the former 97.5% and has led to the conclusion that feature reduction by inspecting densities of single features is not applicable when the used classifier is non-linear and apply a kernel mapping as is the case with the RBF-SVM classifier.

### 3.3. Feature Selection

**Figure 9.**
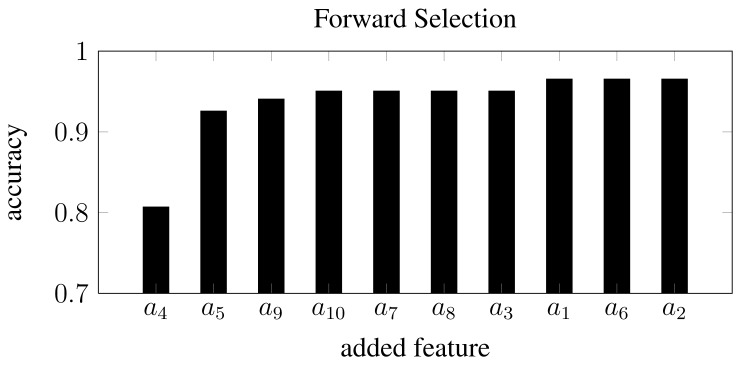
Results of the feature selection process using forward selection. The x tick label specifies the feature that has been added to the subset to the left.

Inspecting single feature distributions is a viable way to get insight into single feature but this method lacks information on correlation. To overcome this, feature selection was conducted by using forward selection. This resulted in 10 feature subsets with an increasing number of features starting from a single feature and resulting in a subset including all features. Forward selection is conducted by first training classifiers with only one feature. The best performing feature is chosen as the first subset. A new iteration is started where this subset of one feature is combined with one of the remaining possible features. In this way, several new subsets of two features are generated and the best performing subset is chosen for the next iteration. This process is repeated until a termination criterion is met or all features have been included. This approach is controlled by correlation since single features that perform well will not necessarily perform well in combination with highly correlated ones. The result of this feature selection process can be seen in Figure 9. By inspecting the subsets one can verify that the first feature selected in the forward feature selection process was the coefficient *a*_4_, which was also the feature that performed best regarding overlapping area in the distribution approach in Subsection 3.2. Furthermore, it can be seen that the performance does not improve much after having included four features. With the subset [*a*_4_, *a*_5_, *a*_9_, *a*_10_], an accuracy of 95% was achieved.

### 3.4. Analysis of Classification Failure

This section will comment on what kinds of errors were observed from the best performing classifiers. Because of the abstract nature of the proposed distance features and the high dimensionality of the feature space, the analysis of failures will be conducted using visual inspection by comparing true class samples and erroneously classified samples.

**Figure 10.**
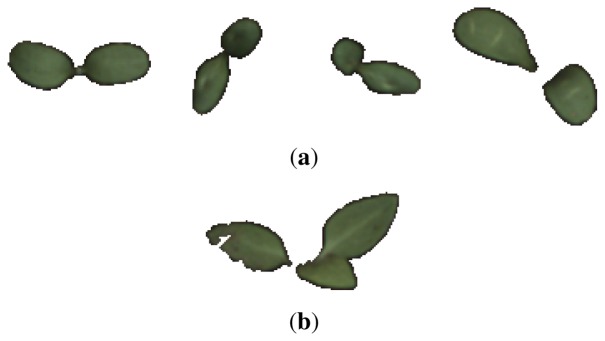
RBF-classification errors. (**a**) Cornflower sample classified as nightshade; (**b**) Nightshade classified as cornflower.

Figure 10 shows the five samples that were erroneously classified by an RBF-SVM classifier. Comparing those to other class samples in Figures 1(a) and 1(b) (see [23] for the full data set) reveals that the samples in Figure 10(a) seems to be either very early in their development stage or in an (for this data set) unusual pose. The sample in Figure 10(b) is in a stage where the two cotyledons have developed and a true leaf is on its way, but in this particular situation the true leaf overlaps one of the germination leaves, changing the object outline dramatically. This is a rare situation in the used data set.

## 4. Discussion and Future Work

This paper has dealt with a data set consisting of plant samples from two species at approximately the same growth stage. The results reported should be seen in this context as it is expected that performance will drop if additional species and growth stages were included. In spite of a limited data set, the results still encourage a further investigation of the proposed feature sets. Other researchers also have reported remarkable recognition accuracies of above 95%, but a comparison tends to be difficult. Comparing results raises several problems. The investigated species often are not the same as the ones used in other studies, which is also the case here. Specifically for this study, only 2 species have been used, but other studies handling additional species tend to group species and also end up with only a few classes such as dicotyledonous and monocotyledonous or crop and weeds. The authors of this paper are convinced that a common database with plant species is needed to enable researchers to test their recognition methods and directly compare results. In other image analysis areas, common databases are available, but it is believed that recognition of plant species is such a special case that methods that perform well on other publicly available data set of objects might not be transferable to plant species recognition. The authors of this paper are planning to create a publicly available database with 14 species of 80 to 100 unique samples of each species at growth BBCH 10 to BBCH 12.

## 5. Conclusions

We have described how to compute two novel sets of closed contour shape features. The feature sets are referred to as RSFS and LPFS. Four approaches to data preprocessing have been investigated. The preprocessing methods are based on the result of a distance transformation. All combinations of data preprocessing and feature generation methods have been tested on a dataset containing samples from two plant species. The test consisted of letting four different classification schemes take the generated feature sets as input. The best performing classifier was a Radial-Basis Function Support Vector Machine classifier taking a LPFS as input feature vector and using the preprocessing data mapping referred to as *D_scaled_*. This led to a classification accuracy of 97.5%. In comparison, the best performing classifier trained and tested with 21 features found in the literature, CFS, obtained a classification accuracy of 92.5%. The results suggest that LPFS can compete with CFS. When conducting feature selection by forward selection, a feature subset of only 4 features resulted in an accuracy of 95%. Further testing is needed to reveal the generality of *LPFS*. Investigating the errors made by the best performing classifier showed that erroneously classified samples belong to sparsely represented regions of the underlying feature space.

## Figures and Tables

**Table 1.** Complexity of preprocessing steps. *q* denotes the number of pixels in an image.

**Task**	**Complexity**
Segmentation	O(q)
Connected component	O(q)

**Table 2.** Complexity of connected component processing steps. *m* is the number of pixels in a connected component. *order* is the polynomial order of the fitted polynomial.

**Method**	**Complexity**
Distance transform	O(m)
Distance sorting	O(m Log(m))
Linear mapping	O(m)
Resampling	O(1)
Polynomial fit	O(m * order)

**Table 3.** Classification results from using 4 classifier models and the CFS.

	**k-NN**	**Naive Bayes**	**linear SVM**	**RBF-SVM**
Accuracy	88.75%	73.75%	92.5%	86.25%
Parameters	k = 3		*C* = 0.279	*sigma* = 706.87 *C* = 0.279
